# Switching to second-generation tyrosine kinase inhibitor improves the response and outcome of frontline imatinib-treated patients with chronic myeloid leukemia with more than 10% of BCR-ABL/ABL ratio at 3 months

**DOI:** 10.1002/cam4.440

**Published:** 2015-03-10

**Authors:** Luis-Felipe Casado, José-Valentín García-Gutiérrez, Isabel Massagué, Pilar Giraldo, Manuel Pérez-Encinas, Raquel de Paz, Joaquín Martínez-López, Guiomar Bautista, Santiago Osorio, María-José Requena, Luis Palomera, María-Jesús Peñarrubia, Carmen Calle, José-Ángel Hernández-Rivas, Carmen Burgaleta, Begoña Maestro, Nuria García-Ormeña, Juan-Luis Steegmann

**Affiliations:** 1 Registro Español de Investigación y Tratamiento de Leucemia Mieloide Crónica (RELMC) [Spanish Registry for the Investigation and Treatment of Chronic Myeloid Leukemia]Madrid, Spain; 2 Servicio de Hematología y Hemoterapia, Hospital Virgen de la SaludToledo, Spain; 3 Servicio de Hematología y Hemoterapia, Hospital Universitario Ramón y CajalMadrid, Spain; 4 Servicio de Hematología y Hemoterapia, Hospital Valle de HebrónBarcelona, Spain; 5 Servicio de Hematología y Hemoterapia, Hospital Universitario Miguel ServetZaragoza, Spain; 6 Servicio de Hematología y Hemoterapia, Hospital Clínico Universitario de Santiago de CompostelaSantiago de Compostela, Spain; 7 Servicio de Hematología y Hemoterapia, Hospital Universitario La PazMadrid, Spain; 8 Servicio de Hematología y Hemoterapia, Hospital 12 de OctubreMadrid, Spain; 9 Servicio de Hematología y Hemoterapia, Hospital Puerta de HierroMajadahonda, Spain; 10 Servicio de Hematología y Hemoterapia, Hospital General Universitario Gregorio MarañónMadrid, Spain; 11 Servicio de Hematología y Hemoterapia, Hospital Universitario Severo OchoaLeganés, Spain; 12 Servicio de Hematología y Hemoterapia, Hospital Lozano BlesaZaragoza, Spain; 13 Servicio de Hematología y Hemoterapia, Hospital Clínico Universitario de ValladolidValladolid, Spain; 14 Servicio de Hematología y Hemoterapia, Hospital General de Ciudad RealCiudad Real, Spain; 15 Servicio de Hematología y Hemoterapia, Hospital Universitario Infanta LeonorMadrid, Spain; 16 Servicio de Hematología y Hemoterapia, Hospital Universitario Príncipe de AsturiasAlcalá de Henares, Spain; 17 Servicio de Hematología y Hemoterapia, Hospital Universitario de la PrincesaMadrid, Spain

**Keywords:** BCR-ABL/ABL ratio, chronic myeloid leukemia, dasatinib, imatinib, nilotinib

## Abstract

Chronic myeloid leukemia patients display heterogeneous responses to imatinib. Survival depends on baseline clinical characteristics (including prognostic scoring systems) and on early response (such as >10% BCR-ABL/ABL ratio at 3 months of therapy). The results of switching to second-generation tyrosine kinase inhibitors (2GTKIs) may contain a bias since, in the majority of these studies, patients who switch treatment due to intolerance or failure are censored or excluded. We analyzed the Spanish Registry data on switching in an intention-to-treat analysis of patients in standard clinical practice. Switching to 2GTKIs improves responses from 45% to 75% of complete cytogenetic response (CCyR) and from 15% to 45% of major molecular response (MMR) in the group without molecular response 1 (MR1) at 3 months and from 70% to 87% in CCyR and from 52% to 87% in MMR in the group with MR1. The final response rate is poorer in the group with no MR1 at 3 months. Nevertheless, the differences in the rates of response were not translated into differences in major events (transformations or deaths), and the final progression-free survival and overall survival were similar.

## Introduction

The introduction of tyrosine kinase inhibitors (TKIs) has proved to be a major advance in the management of patients with chronic myeloid leukemia in chronic phase (CML-CP). Imatinib therapy has radically changed the prognosis for patients with CML, and most patients enjoy a near-normal life expectancy 1. Recently, efforts have been made to define molecular markers, or clinical milestones, that predict the course of patient outcomes more reliably than cytogenetics but, to-date, such findings are not fully reproducible. CML patients display heterogeneous response to therapy, with survival often depending on baseline clinical characteristics. Until recently, the stratification was made according to two scores developed in CML patients treated with chemotherapy 2, and interferon (Euro) 3. In 2011, the European Leukaemia Net (ELN) evaluated more than 2000 patients with early chronic phase (ECP) CML treated with front-line imatinib; the objective was to develop a new prognostic scoring system: the European Treatment and Outcome Study (Eutos) score 4. Since the publication of the system, however, there have been conflicting reports on the predictive value of the Eutos score. The Spanish RELMC (Registro Español de Investigación y Tratamiento de Leucemia Mieloide Crónica) is a hospital-based registry, which includes only out-of-study patients. Monitoring and treatment are at the physician’s discretion, and do not reflect any clinical pathway. There is no common protocol and no common recommendations since the aim of the Registry is to monitor the current practice of CML management in Spain.

Recently, a cut-off value of BCR-ABL/ABL ratio of >10% at 3 months has been proposed to discriminate patients with a poorer outcome when treated with imatinib 5, dasatinib 6, nilotinib 7, and bosutinib 8. The aims of the present study were to discriminate the influence of this response variable on outcomes in CML patients, and to assess the value of preclinical scores (Sokal, Euro, and Eutos) in this model. In addition, data are limited on how this outcome is affected by the change to second-generation TKI (2GTKI). The switching to 2GTKIs introduces a bias in the majority of studies because patients who switch treatment due to intolerance or failure are censored and, as such, “increasing” the probability of response in their group 9. To avoid this bias, the variable “time-to-imatinib failure” was introduced into our outcomes analysis.

## Methods

### Patients and therapy

We retrospectively analyzed 374 patients with CML in first CP (treated with imatinib as first choice, and outside of clinical trials). Table1 summarizes patients’ characteristics. Complete hematologic response (CP), complete cytogenetic response (CCyR), and major molecular response (MMR) were defined using conventional criteria. Follow-up and treatment decisions were decided by hematologists according to their own clinical judgment, that is, no preset protocol. Cytogenetic or molecular data were included until the time that imatinib treatment was discontinued, for whatever reason, or at last follow-up.

**Table 1:** Patient characteristics

	All patients	Patients with RTQ-PCR at 3 months
*N*	374	156/374 (42%)
Duration imatinib (days)	19 (1–180)	19 (1–180)
Male/Female	229 (61%)/145(39%)	93 (60%)/63(40%)
Age, years (range)	52 (15–88)	54.2 (15–86)
Sokal index		
Low	138 (39%)	62 (40%)
Intermediate	172 (48%)	78 (50%)
High	44 (12%)	15 (10%)
Euro index		
Low	170 (48%)	82 (53%)
Intermediate	165 (47%)	65 (42%)
High	19 (5%)	6 (5%)
Eutos index		
Low	349 (91%)	138 (91%)
High	30 (9%)	14 (9%)

RTQ-PCR, real-time quantitative polymerase chain reaction.

### Detection of *BCR-ABL1* transcripts

*BCR-ABL1* transcripts were measured in venous blood samples taken at 12-week intervals, using real-time quantitative polymerase chain reaction (RTQ-PCR) as described previously 10. Results were expressed as percentage ratios relative to an *ABL1* internal control. *BCR-ABL1* transcript samples were not centralized but all RTQ-PCR analyses were performed in the same laboratory for each patient during follow-up. In our registry of patients treated with frontline imatinib, 156 (41.7%) of 374 had a molecular ratio of BCR-ABL/ABL determined at 3 months. During follow-up, 43 of 156 patients discontinued imatinib and had switched to receive dasatinib (*n* = 30), or nilotinib (*n* = 13), while five patients were treated with two TKI’s; dasatinib–nilotinib in sequence (*n* = 4) and nilotinib–dasatinib in sequence (*n* = 1).

### Statistical methods

For the purpose of this study, and for clarity, we used MR1 (molecular response 1) to represent patients who achieved a BCR-ABL/ABL ratio ≤10%.

Those patients with treatment failure at 3 months, as defined by ELN06, were excluded from the statistical analyses. Probabilities of overall survival (OS), progression-free survival (PFS), and time-to-imatinib failure (TIF) were calculated using the Kaplan–Meier method. In TIF, an event was defined as a progression to advanced phase, death, or imatinib discontinuation (for whatever reason). For PFS, the corresponding events were death, progression to accelerated phase (AP), or blast crisis (BC) 11. The probabilities of cytogenetic and molecular responses were calculated using the cumulative incidence procedure. The log-rank test or a Cox regression model was used to compare OS, PFS, and TIF. Univariate and multivariate analyses were performed in accordance with standard methods; variables with *P* < 0.10 in univariate analyses were entered in the multivariate analysis. Unless stated in the text, all the analyses were performed on an intention-to-treat basis. Differences between groups were evaluated by the chi-square test and Mann–Whitney *U* test for categorical and continuous variables, respectively. Survival probabilities were estimated by the Kaplan–Meier method and compared with the log-rank test. Univariate and multivariate analyses were performed to identify potential factors predicting early cytogenetic responses. Multivariate analysis of response used the logistic regression model and, for time-to-event outcomes, we used the Cox proportional hazard regression.

## Results

The median follow-up in the current series of patients was 54 months (range: 1–174 months). The distributions of BCR-ABL/ABL ratios at 3 months according to Sokal, Euro, and Eutos are summarized in Table2. Sokal, Euro, or Eutos risk scores were not significantly associated with cut-off points, but the proportion of patients with ratio >10% was higher in Sokal and Euro high-risk groups.

**Table 2:** Index score distributions according to BCR-ABL/ABL ratio at 3 months

	Sokal index			Euro index			Eutos index	
Risk score	Low	Intermediate	High	Low	Intermediate	High	Low	High
≤10%106/156 (68%)	45/62 (73%)	53/78 (68%)	8/15 (53%)	59/82 (72%)	42/65 (65%)	3/6 (50%)	93/138 (67%)	10/14 (71%)
>10%50/156 (32%)	17/62 (27%)	25/78 (32%)	7/15 (47%)	23/82 (28%)	23/65 (35%)	3/6 (50%)	45/138 (33%)	4/14 (29%)
*P*-value	0.353			0.402			0.758	

### Response to imatinib

The rate of best CCyR and MMR reached with imatinib therapy were 81% and 75.4%, respectively. The data are summarized in Table3. The percentages of subsequent best CCyR and MMR in the group with MR1 at 3 months were significantly higher than those of patients not achieving MR1 (95% confidence interval [CI]: 92–88% vs. 77–58%; *P* < 0.001).

**Table 3:** Best cytogenetic and molecular response with imatinib treatment alone segregated according BCR-ABL/ABL ratio at 3 months; outcome in patients who switched to 2GTKI in both groups

	Best response with imatinib		Best response with imatinib alone^*^ or imatinib + 2GTKIs (switch group)					Best response (all treatments)		
BCR-ABL/ABL ratio at 3 months	CCyR	MMR	Switch 2GTKIs	CCyR		MMR		Events (AP, BC, or deaths)	CCyR	MMR
				Before switch	After switch	Before switch	After switch			
≤10%106/156 (68%)	94/106 (89%)	85/106 (80%)	Yes: 23/106	16/23^*^ (70%)	20/23 (87%)	12/23^*^ (52%)	20/23 (87%)	3/23 (13%)	98/106 (92%)	93/106 (88%)
			No: 83/106	78/83^*^ (94%)		73/83^*^ (88%)		4/83 (5%)		
More than 10%50/156 (32%)	31/47 (66%)	23/49 (47%)	Yes: 20/50	9/20^*^ (45%)	15/20 (75%)	3/20 (15%)	9/20 (45%)	2/20 (10%)	37/48 (77%)	29/50 (58%)
			No: 30/50	22/28^*^ (79%)		20/30^*^ (67%)		2/30 (7%)		
*P*-value	<0.0001	<0.0001							0.007	<0.0001

2GTKI, second generation tyrosine kinase inhibitor; CCyR, complete cytogenetic response; MMR, major molecular response; AP, accelerated phase; BC, blast crisis.

### Time-to-imatinib failure

During follow-up (median 80 months), 43 patients had their therapy changed to a 2GTKI. Of these, 30 were assigned to dasatinib, 13 to nilotinib, while five patients were treated with two TKIs: dasatinib–nilotinib in sequence (*n* = 4) and nilotinib–dasatinib in sequence (*n* = 1). The outcome was an 8-year probability of imatinib failure-free survival of 60%. In the group with MR1 at 3 months, 23 (22%) of 106 patients switched to 2GTKIs because of lack of efficacy (*n* = 4), intolerance (*n* = 10), and loss of response (*n* = 9). The distribution was different in the group that did not archive MR1, that is, intolerance (*n* = 8), loss of response (*n* = 1), and lack of efficacy (*n* = 11) for a total of 20 (40%) of 50 patients (Table4).

**Table 4:** Causes and time-of-switch to 2GTKIs

BCR-ABL/ABL ratio at 3 months	Switch 2GTKIs	Causes			Time to switch (years)		
		Intolerance	No optimal response	Loss of response	1	2	3
≤10%106/156 (68%)	Yes: 23/106 (22%)	10/23 (43%)	4/23 (17%)	9/23 (39%)	10/23 (43%)	5/23 (22%)	8/23 (35%)
	No: 83/106 (88%)						
More than 10%50/156 (32%)	Yes: 20/50 (40%)	8/20 (40%)	11/20 (55%)	1/20 (5%)	8/20 (40%)	7/20 (35%)	5/20 (25%)
	No: 30/50 (60%)						
*P*-value	<0.017	0.008					

2GTKI, second generation tyrosine kinase inhibitor.

**Figure 1:**
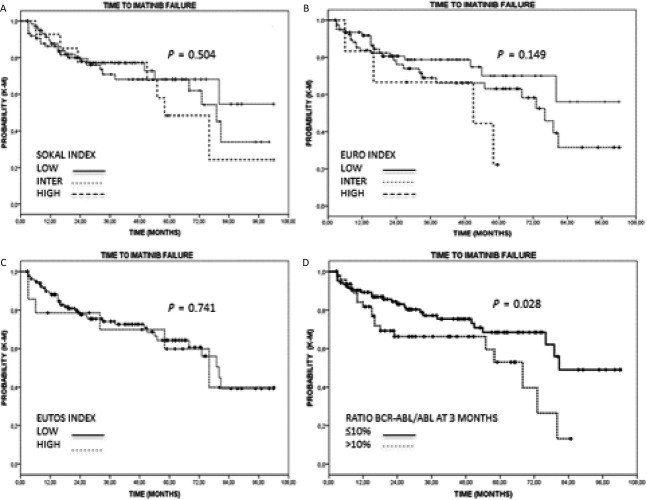
Time-to-imatinib failure segregated according to the molecular response at 3 months or risk group. (A) TIF by Sokal risk index; (B) TTIF by Euro risk index; (C) TIF by Eutos risk index; (D) TIF by molecular response at 3 months. TIF, time-to-imatinib failure; Eutos, European Treatment and Outcome Study.

In univariate analysis, only having MR1 at 3 months was significantly associated with higher probability of imatinib treatment failure (long-rank test *P* = 0.026) (Fig.1) and this variable was the only one associated with poorer outcome in multivariate analysis (hazard ratio: 1.91; 95% [CI]: 1.619–2.209; *P* = 0.027) (Table5).

**Table 5:** Multivariate analysis of factors predictive of time-to-imatinib failure

Covariates	*β*	SE	Wald	*df*	*P*	HR
Gender	−0.060	0.295	0.041	1	0.839	0.942
Sokal risk group	0.116	0.277	0.175	1	0.675	1.123
Euro risk group	0.306	0.312	0.964	1	0.326	1.358
Eutos risk group	−0.172	0.470	0.134	1	0.714	0.842
Ratio BCR-ABL/ABL at 3 months (>10% vs. ≤10%)	0.649	0.295	4.859	1	0.027	1.914

SE, standard error; df, degrees of freedom; HR, hazard ratio.

### Response rate to 2GTKIs after switching

**Figure 2:**
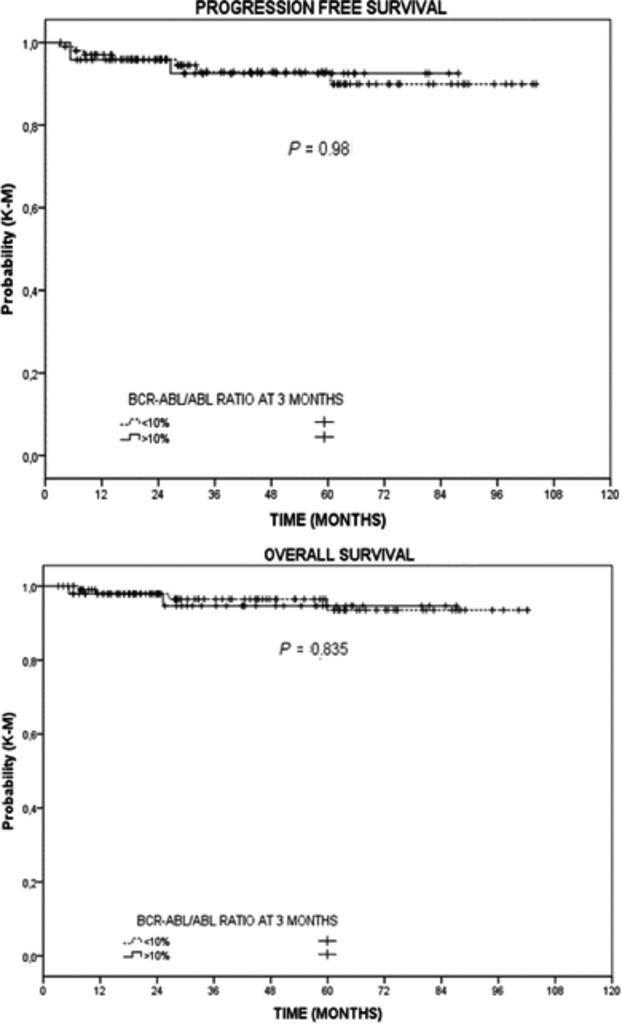
Progression-free survival (PFS) or overall survival (OS) segregated according to the molecular response at 3 months.

Switching to 2GTKIs improves the mean rates of response in both groups (from 45% to 75% of CCyR and from 15% to 45% of MMR in the group without MR1 at 3 months and from 70% to 87% in CCyR and from 52% to 87% in MMR in the group with MR1) (Table3). This improvement in mean response rate with switching was reflected in the best response rates obtained in the groups; the CCyR and MMR were 92% and 88% in the group with MR1 at 3 months and the corresponding values were 77% and 58% in the non-MR1 group (*P* < 0.001). Nevertheless, the rates of response were not translated into differences in major events (transformations or deaths) (Table6). The final PFS and OS were similar in both groups (Fig.2).

**Table 6:** Rate of events: accelerated phase (AP), blast crisis (BC), or deaths in each group

BCR-ABL/ABL ratio at 3 months	AP	BC	Deaths	Deaths or transformations
≤10%106/156 (68%)	2/106 (2%)	1/106 (1%)	4/106 (4%)	7/106 (7%)
>10%50/156 (32%)	0/47 (0%)	1/50 (2%)	3/50 (6%)	4/50 (8%)
*P*-value	0.084	0.584	0.531	0.751

## Discussion

The observation of prognostic significance of response to TKI after 3 months of the start of therapy has triggered a debate on the optimal management of these patients. Currently, there are no data available from prospective studies suggesting that molecular response-driven change in therapy among these patients alters the final outcome. The NCCN has recommended that these patients should be offered a change in therapy 12, but ELN2013 considered this status as a need for caution 13.

Despite the prognostic importance of MR1 at 3 months, the actual incidence of MR1 has not been clearly established in real practice. When we analyzed prospective studies comparing imatinib with 2GTKIs (ENESTND, DASISION, and BELA trials) the proportion of patients with no MR1 at 3 months in the imatinib group was similar (33.3%, 36%, and 34.5% in the three trials, respectively) 6–8. We have seen a similar rate (32%) in our study, confirming that at least one third of patients will fail to respond to the treatment (assuming that the proportion of patients at high-risk is comparable with our study).

Our population sample had a lower percentage of patients with a high-risk index (Sokal, Eutos, or Euro score) at diagnosis than that reported in other series. These scores seem to be good predictors of response at 3 months. Patients with high-risk score at baseline had a higher probability of failing to achieve MR1 at 3 months. Moreover, despite the negative influence of the diagnostic score, the prognostic weight does not seem to be maintained if patients achieved MR1 at 3 months; and this response is the only variable to remain significant in the multivariate analysis.

All changes in treatment decisions were made according to the ELN recommendations; most patients changed at different times and for different reasons 14,15. However, no patient in this study switched before 3 months (we excluded patients from the statistical analysis who were considered failures according to the ELN06 recommendations at 3 months), and many of them had high-risk scores. To the best of our knowledge, our study is the first to describe the actual treatment given to the patients when BCR-ABL/ABL ratio at 3 months is the variable of interest. Other studies have analyzed the outcomes with respect to this ratio, but they have not reported the influence of the change in therapy, and they do not provide information about the specific treatment given as second line. The only exception is the TIDEL II study, the intervention in which depends on specific molecular markers 16. We can conclude from our results that these changes in therapy (at different times depending on the different recommendations) have enabled a significant number of patients to improve their response rates. However, the final outcomes in terms of PFS and the OS are similar in all patients (despite the poor initial forecast in patients with no MR1 at 3 months).

Our results show that the 10% threshold at 3 months discriminates, in a statistically significant manner, the probability of obtaining CCyR and MMR subsequently, not only when first-line imatinib is considered but also when all lines of TKI treatment are taken into account. However, this did not translate in to differences in OS or in PFS. This lack of difference in survival outcomes between the two groups has not been concordant in all recent studies. Hanfstein et al. 17 reported significantly better PFS and OS probabilities in patients who achieved *MCyR* or BCR-ABL/ABL ratio ≤10% with 3 months of imatinib therapy, while Marin et al. 5 reported that achieving BCR-ABL-ABL ratio ≤9.84% at 3 months was linked to higher probabilities of CCyR, MMR, MR, PFS, and OS in patients treated with imatinib. In all arms of the ENESTND trial, early molecular response failure (>10% BCR-ABL/ABL ratio at 3 months of therapy) was associated with lower rates of molecular response, an increased risk of progression, and lower OS compared to those achieving early molecular response 7. In the DASISION study 6, early molecular response failure was associated with lower rates of molecular response, an increased risk of progression, and lower PFS and OS.

None of these studies have described the treatment of patients after imatinib failure. The influence of switching treatment on the response and on the survival outcomes were not explored (data were not reported and patients censored for response). Furthermore, the influence of early treatment change was not analyzed. As such, it is important to highlight that, in the ENESTND trial, early progressions were common in the imatinib arm (7 of the 15 patients progressed between 3 and 6 months, representing a rate of 2.4% in 3 months). In the IRIS trial, the first annual rate of progression to AP and BC 11 was 1.5% 18.

Conversely, in a recent analysis of all patients from clinical trials, the MD Anderson group reported the same lack of difference we found in our study. They analyzed early cytogenetic response (at 3 and 6 months) and observed differences in time-to-failure, but not in OS 19. In their study, only one patient progressed to BC at 3 months and no transformations occurred between 3 and 6 months. None of the patients had switched treatment in that period while, in our study, up to 40% switched within the first year.

In our series, switching to 2GTKI, although associated with significant improvement in response rates, does not appear to overcome the poor outcome prediction of the 10% cut-off at 3 months; the response rate is poorer in the group with no MR1 at 3 months. The reason for lack of translation of the difference in responses into probabilities of better PFS or OS is not clear. It could reflect the efficacy of the treatment in third line and beyond. The explanation is hampered by the reason(s) for switching being different in the two groups, that is, more secondary resistance in the MR1 group, more primary resistance in the group without MR1 at 3 months.

In summary, our results confirm (in real-life practice within the setting of an intention-to-treat analysis and with a long follow-up) that not obtaining a BCR-ABL/ABL ratio of ≤10% at 3 months is a warning sign because it compromises the potential response to treatment even when switching to a 2GTKI. This emphasizes the importance of evaluating new treatment approaches when this threshold is not met.

## References

[b1] Hasford J, Pfirrmann M, Hochhaus A (2005). How long will chronic myeloid leukemia patients treated with imatinib mesylate live?. Leukemia.

[b2] Sokal JE, Cox EB, Baccarani M, Tura S, Gomez GA, Robertson JE (1984). Prognostic discrimination in “good-risk” chronic granulocytic leukemia. Blood.

[b3] Hasford J, Pfirrmann M, Shepherd P, Guilhot J, Hehlmann R, Mahon FX (2005). The impact of the combination of baseline risk group and cytogenetic response on the survival of patients with chronic myeloid leukemia treated with interferon alpha. Haematologica.

[b4] Hasford J, Baccarani M, Hoffmann V, Guilhot J, Saussele S, Rosti G (2011). Predicting complete cytogenetic response and subsequent progression-free survival in 2060 patients with CML on imatinib treatment: the EUTOS score. Blood.

[b5] Marin D, Ibrahim AR, Lucas C, Gerrard G, Wang L, Szydlo RM (2012). Assessment of BCR-ABL1 transcript levels at 3 months is the only requirement for predicting outcome for patients with chronic myeloid leukemia treated with tyrosine kinase inhibitors. J. Clin. Oncol.

[b6] Jabbour E, Kantarjian HM, Saglio G, Steegmann JL, Shah NP, Boque C (2014). Early response with dasatinib or imatinib in chronic myeloid leukemia: 3-year follow-up from a randomized phase 3 trial (DASISION). Blood.

[b7] Hughes TP, Saglio G, Kantarjian HM, Guilhot F, Niederwieser D, Rosti G (2014). Early molecular response predicts outcomes in patients with chronic myeloid leukemia in chronic phase treated with frontline nilotinib or imatinib. Blood.

[b8] Brummendorf TH, Kantarjian HM, Gambacorti-Passerini C, Guilhot F, Akard L, Doshi V (2012). Assessment of early molecular response as a predictor of long-term clinical outcomes in the phase 3 BELA study. ASH Annual Meeting Abstracts.

[b9] Guilhot J, Baccarani M, Clark RE, Cervantes F, Guilhot F, Hochhaus A (2012). Definitions, methodological and statistical issues for phase 3 clinical trials in chronic myeloid leukemia: a proposal by the European LeukemiaNet. Blood.

[b10] Hughes T, Deininger M, Hochhaus A, Branford S, Radich J, Kaeda J (2006). Monitoring CML patients responding to treatment with tyrosine kinase inhibitors: review and recommendations for harmonizing current methodology for detecting BCR-ABL transcripts and kinase domain mutations and for expressing results. Blood.

[b11] Sacha T, Hochhaus A, Hanfstein B, Muller MC, Rudzki Z, Czopek J (2003). ABL-kinase domain point mutation as a cause of imatinib (STI571) resistance in CML patient who progress to myeloid blast crisis. Leuk. Res.

[b12] O’Brien S, Berman E, Moore JO, Pinilla-Ibarz J, P Radich J, Shami PJ (2011). NCCN Task Force report: tyrosine kinase inhibitor therapy selection in the management of patients with chronic myelogenous leukemia. J. Natl. Compr. Canc. Netw.

[b13] Baccarani M, Deininger MW, Rosti G, Hochhaus A, Soverini S, Apperley JF (2013). European LeukemiaNet recommendations for the management of chronic myeloid leukemia: 2013. Blood.

[b14] Baccarani M, Saglio G, Goldman J, Hochhaus A, Simonsson B, Appelbaum F (2006). Evolving concepts in the management of chronic myeloid leukemia: recommendations from an expert panel on behalf of the European LeukemiaNet. Blood.

[b15] Baccarani M, Cortes J, Pane F, Niederwieser D, Saglio G, Apperley J (2009). Chronic myeloid leukemia: an update of concepts and management recommendations of European LeukemiaNet. J. Clin. Oncol.

[b16] Yeung DT, Osborn MP, White DL, Branford S, Kornhauser M, Slader C (2012). Early switch to nilotinib does not overcome the adverse outcome for CML patients failing to achieve early molecular response on imatinib, despite excellent overall outcomes in the TIDEL II trial. ASH Annual Meeting Abstracts.

[b17] Hanfstein B, Muller MC, Hehlmann R, Erben P, Lauseker M, Fabarius A (2012). Early molecular and cytogenetic response is predictive for long-term progression-free and overall survival in chronic myeloid leukemia (CML). Leukemia.

[b18] Hochhaus A, O’Brien SG, Guilhot F, Druker BJ, Branford S, Foroni L (2009). Six-year follow-up of patients receiving imatinib for the first-line treatment of chronic myeloid leukemia. Leukemia.

[b19] Nazha A, Kantarjian H, Jain P, Romo C, Jabbour E, Quintas-Cardama A (2013). Assessment at 6 months may be warranted for patients with chronic myeloid leukemia with no major cytogenetic response at 3 months. Haematologica.

